# Early primary biliary cholangitis is characterised by brain abnormalities on cerebral magnetic resonance imaging

**DOI:** 10.1111/apt.13797

**Published:** 2016-09-08

**Authors:** V. P. B. Grover, L. Southern, J. K. Dyson, J. U. Kim, M. M. E. Crossey, M. Wylezinska‐Arridge, N. Patel, J. A. Fitzpatrick, A. Bak‐Bol, A. D. Waldman, G. J. Alexander, G. F. Mells, R. W Chapman, D. E. J. Jones, S. D. Taylor‐Robinson

**Affiliations:** ^1^ Liver UnitDivision of Diabetes, Endocrinology and MetabolismDepartment of MedicineImperial College LondonLondonUK; ^2^ Robert Steiner MRI UnitImaging Sciences DepartmentMRC Clinical Sciences CentreImperial College LondonLondonUK; ^3^ Institute of Cellular MedicineNewcastle UniversityNewcastle‐upon‐TyneUK; ^4^ Cambridge Hepatobiliary ServiceAddenbrookes Hospital. Hills RoadCambridgeUK; ^5^ Nuffield Department of MedicineOxford UniversityJohn Radcliffe HospitalOxfordUK

## Abstract

**Background:**

Brain change can occur in primary biliary cholangitis (PBC), potentially as a result of cholestatic and/or inflammatory processes. This change is linked to systemic symptoms of fatigue and cognitive impairment.

**Aim:**

To identify whether brain change occurs early in PBC. If the change develops early and is progressive, it may explain the difficulty in treating these symptoms.

**Methods:**

Early disease brain change was explored in 13 patients with newly diagnosed biopsy‐proven precirrhotic PBC using magnetisation transfer, diffusion‐weighted imaging and ^1^H magnetic resonance spectroscopy. Results were compared to 17 healthy volunteers.

**Results:**

Cerebral magnetisation transfer ratios were reduced in early PBC, compared to healthy volunteers, in the thalamus, putamen and head of caudate with no greater reduction in patients with greater symptom severity. Mean apparent diffusion coefficients were increased in the thalamus only. No ^1^H magnetic resonance spectroscopy abnormalities were seen. Serum manganese levels were elevated in all PBC patients, but no relationship was seen with imaging or symptom parameters. There were no correlations between neuroimaging data, laboratory data, symptom severity scores or age.

**Conclusions:**

This is the first study to be performed in this precirrhotic patient population, and we have highlighted that neuroimaging changes are present at a much earlier stage than previously demonstrated. The neuroimaging abnormalities suggest that the brain changes seen in PBC occur early in the pathological process, even before significant liver damage has occurred. If such changes are linked to symptom pathogenesis, this could have important implications for the timing of second‐line‐therapy use.

## Introduction

Patients with the autoimmune cholestatic liver disease primary biliary cholangitis [formerly primary biliary cirrhosis (PBC)] frequently exhibit both central nervous system (CNS) symptoms and neurophysiological and functional CNS abnormality. Fatigue is a significant problem in patients with PBC, and although partly peripheral in origin, there appears to be a central component associated with sleep disturbance and autonomic dysfunction.^1^, ^2^, ^3^, ^4^ Patients with PBC also describe subtle cognitive impairment (particularly relating to concentration and memory) which can lead to significant functional impairment,^5^ a phenomenon that has been associated with defective central corticotropin‐releasing hormone neurotransmission and TReg inhibition in cholestatic animal models.^6^, ^7^ Central fatigue and cognitive impairment in PBC remain un‐responsive to any form of current drug treatment. Furthermore, recent data from the large UK‐PBC patient cohort have suggested that the severity of both fatigue and cognitive symptoms post‐transplant in PBC is similar to that seen in the un‐transplanted population, raising the possibility that the process responsible for CNS abnormality is not reversed by transplantation.^8^ Prospective studies, albeit in smaller patient numbers, have confirmed ongoing fatigue in post‐transplant patients, with a severity similar to that seen in the un‐transplanted PBC population.^9^ The apparent lack of change in CNS symptomology in precirrhotic PBC following liver transplantation highlights the need for improved therapy earlier in the disease course to change its natural history.

Therapeutics in PBC is in the process of being transformed by the advent of effective second‐line therapy. Primary therapy with ursodeoxycholic acid (UDCA) is effective in the majority of people and 50% of patients unresponsive to UDCA have been shown to respond to the first of the second‐line agents, obeticholic acid (OCA), a Farnesoid X receptor (FXR) agonist.^10^, ^11^ Combination therapy with UDCA and fenofibrate has also been proposed for patients who exhibit an incomplete UDCA response, however, high quality trial data are currently lacking.^12^, ^13^ The proposed paradigm for OCA at present is to restrict its use to patients who have demonstrated lack of response to UDCA. When used in this way, the trials of OCA show no benefit in terms of fatigue or cognitive impairment in PBC patients, and this remains a frustrating aspect of the otherwise very promising therapy profile for this agent.^10^ One possible explanation for the lack of benefit on CNS symptoms of an otherwise highly effective agent could be that brain change in PBC (which has already been demonstrated to be irreversible following transplantation) may be something which actually develops from early in the disease process, rather than being a late‐stage phenomenon. It may, therefore, be that the current treatment paradigm for OCA mitigates against beneficial effect on brain change. At present, however, the data regarding early PBC, and the extent to which CNS abnormality is present, and might thus be reasonably targeted by more effective anticholestatic therapy, are limited. All published studies of organic brain change in PBC have been limited to advanced‐stage cirrhotic patients. The study of patients with early disease is warranted to explore the hypothesis that brain change starts early in the disease; a finding which if confirmed would warrant a change in proposed treatment paradigms.

The basal ganglia and the globus pallidus in particular are key regions of the brain with associated pathophysiology in a variety of conditions ranging from movement disorders, such as Parkinson's disease^14^ to chronic hepatitis C, and in manganese workers, who have been exposed to industrial pollution.^15^, ^16^, ^17^ It has been previously hypothesised that disrupted activity in these areas of the brain leads to decreased motivation in these conditions, perceived by the individual as fatigue.^18^ Furthermore, the basal ganglia and the globus pallidus in particular have been shown to be susceptible to manganese accumulation associated with cholestasis of any cirrhotic state, while patients with chronic liver disease exhibit pallidal hyperintensity on T_1_‐weighted magnetic resonance imaging (MRI), similar to that seen in hypermanganesaemic states, such as chronic parenteral nutrition administration and manganese toxicity from industrial exposure.^19^, ^20^

The main investigative modality for CNS abnormalities in PBC is, therefore, cerebral magnetic resonance imaging (MRI) as manganese is a relaxation agent affecting both T_1_ and T_2_ parameters.^18^ Imaging studies performed to date have identified the presence of white matter lesions in the brains of PBC patients and there is objective evidence of a cerebral auto‐regulation abnormality.^5^, ^20^

Furthermore, magnetisation transfer (MT) sequences, in patients with PBC who have established cirrhosis, have defined abnormalities in the basal ganglia which have been attributed either to manganese accumulation or to changes in brain water content.^18^, ^20^ While MT data are simple to acquire in the brain, there are multiple factors that may influence their value and affect the interpretation in PBC. The MT effect is based on comparing signal from water bound to intracellular macromolecules, compared to unbound or ‘free’ intracellular water and determining the shift between these compartments.^21^ The MT effect is thus determined by (i) the physico‐chemical environment of ‘free’ or unbound intracellular water molecules and (ii) the concentration of intracellular macromolecules which may bind the free water.^22^, ^23^, ^24^ If manganese deposition is present, this may also have an effect on magnetisation transfer ratios. Furthermore, it may be anticipated that the magnetisation transfer ratio (MTR) may change as a result of natural ageing processes, apart from pathological disease processes.^20^ Unpicking the various factors responsible for changes observed is difficult and we therefore took a multiparametric imaging approach to define abnormalities more precisely than has been done previously.

All previous MRI studies in PBC have been restricted, however, to patients with advanced disease and established cirrhosis. The aim of the present study was to build on previous work to explore MRI change in the brains of PBC patients, extending the previous studies using magnetisation transfer MRI sequences to newly diagnosed patients with early stage disease. In the current study, we have also applied other methodologies to PBC for the first time at 3 Tesla (T).^18^ Diffusion‐weighted imaging (DWI) allows the investigator to probe the tissue structure at the microscopic level, by quantifying the motion of water molecules. Data obtained may infer changes in intra‐ or intercellular hydration, or changes to the structural integrity of neuronal bundles. The combination of MT and diffusion‐weighted imaging may offer further insight into the pathophysiology in PBC.

We hypothesised that (i) 3T MRI may more accurately define changes in cerebral magnetisation transfer ratios with its inherent signal‐to‐noise advantage in patients with early stage precirrhotic PBC; (ii) the combination of MT imaging, diffusion‐weighted imaging and proton magnetic resonance spectroscopy (^1^H MRS) may better define the etiology of any MR detectable abnormality in patients with precirrhotic PBC and (iii) MR parameters may correlate with manganese levels and/or fatigue data. The data from this study shed light on the genesis of brain injury in PBC, suggesting that change is in fact present from early in the disease process, and supporting the concept that a change in the treatment paradigm to using highly effective therapy early in the disease course is logical.

## Patients and methods

### Patient groups

Thirteen female patients (mean age 57 years, range 34–65) with stage I or II PBC on diagnostic biopsy were recruited from the out‐patient departments at the John Radcliffe Hospital, Oxford and Freeman Hospital, Newcastle within 6 months of that diagnostic biopsy. Seventeen healthy volunteers (11 women and 6 men) with a mean age of 49.8 years (range 40–64), were recruited by open advertisement to staff members and visitors to Imperial College Healthcare Trust, to provide normative control data for MR imaging. None of the healthy volunteers reported any significant medical history.

Patient inclusion criteria were: (i) age 18–65 years; (ii) a liver biopsy consistent with stage I or II precirrhotic PBC; (iii) no evidence of cirrhosis on clinical examination, liver biopsy, laboratory data or imaging; (iv) clinical stability and (v) ability to give informed consent. Medical exclusion criteria for both groups included: (i) history of cerebrovascular disease; (ii) type I diabetes, or type II diabetes with macrovascular complications; (iii) current excessive alcohol consumption (UK National safe drinking limits: 30 g and 20 g per day for men and women, respectively); (iv) current intravenous drug usage; (v) renal impairment (creatinine >150 mmol/L) and (vi) psychoactive drugs or a history of major psychoses.

During the assessment, all patients completed the validated PBC‐40 quality of life measure^25^ and blood tests were taken to assess liver function tests, renal function, full blood count, coagulation studies and serum manganese levels. The latter were processed at the trace metals laboratory at Charing Cross Hospital, Imperial College Healthcare Trust, London, UK.

### MR imaging

Cerebral MRI was performed on a 3T Philips Intera MR system (Philips, Best, the Netherlands). Standard volumetric T_1_‐weighted sequences were performed with a three‐dimensional (3D) imaging sequence: echo time (TE) 3.8 ms, repetition time (TR) 256 ms, number of signal averages (NSA) = 1, 256 image matrix, 25 cm field of view (FOV) and 2.0 mm slice thickness. T_2_‐weighted sequences were performed to exclude structural brain pathology, with the following sequence parameters: TE 80 ms, TR 3000 ms, 2 NSA, image matrix of 230, 23 cm FOV, and 3.0 mm slice thickness. DWI was obtained in 15 directions of sensitisation using single‐shot echo planar imaging (TR 12555 ms, TE 51 ms, slice thickness 2 mm, 2 NSA, *b* = 1000 s/mm^2^). A SENSE factor of 2 was used to reduce image distortion. A 15 direction sequence was also used. MT was obtained using a two‐dimensional gradient‐echo pulse sequence (TR 54.7 ms, TE 3.75 ms, flip angle 15 degrees, slice thickness 2 mm, 1 NSA) with 20 slices positioned over the basal ganglia. ^1^H MRS was acquired using a SENSE headcoil and a short echo time PRESS sequence (TR 2000 ms, TE 36 ms, NSA 64), with volumes of interest of 15 × 15 × 15 mm placed in the left basal ganglia. The sequence was performed three times to give a total NSA of 192.

### MRI analysis

Magnetisation transfer ratio (MTR) maps were calculated, using ImageJ version 1.32j, (www.imagej.nih.gov) with the formula MTR = 100(SI_0_−SI_RF_)/SI_0_, where SI_RF_ is the signal intensity in the image employing an off‐resonance radiofrequency pulse and SI_0_ the signal intensity in the initial proton density image. Regions of interest (ROIs) were drawn around the: (i) frontal white matter; (ii) head of caudate; (iii) putamen; (iv) globus pallidus and (v) thalamus, bilaterally. The same area of ROI was used for each brain region between subjects. The pallidal index (PI) was calculated by the ratio of the left/right averaged signal intensity in the globus pallidus, to the averaged signal intensity of frontal white matter on T_1_‐weighted imaging multiplied by 100.^26^ Signal intensities were measured using ROIs drawn version 1.32j (www.imagej.nih.gov).

Apparent diffusion coefficient (ADC) and fractional anisotropy (FA) maps were calculated using DTI Studio version 2.1 (www.dsi-studio.labsolver.org). Apparent diffusion coefficient and fractional anisotropy values were recorded from specific regions of interest (ROI) in the genu, body and splenium of the corpus callosum. These areas were chosen as they were anatomically highly conspicuous and therefore easily defined on this imaging sequence. A standardised area of ROI was used for the individual ROIs between different subjects.

MR spectra were analysed by two observers (MW and LS), blinded to the clinical status of the patients. Peak areas were measured for choline (Cho), creatine (Cr), myo‐inositol (mI) and N‐acetylaspartate (NAA), using the Advanced Magnetic RESonance (AMARES) algorithm included in the MRUI software package (www.mrui.uab.es), in the time domain. Peak area ratios for NAA/Cr, Cho/Cr and mI/Cr were then calculated.

### Statistical methods

Data were tested for normality using the Shapiro–Wilk test. Between‐group comparisons were made with the Mann–Whitney *U* test. Correlations were made with the Spearman rank test. Tests of significance were two‐tailed. Statistical analyses were performed using spss version 16 (IBM SPSS Statistics for Windows, Version 22.0. Armonk, NY: IBM Corp). Where multiple brain regions were analysed, a multiple correction factor of n‐1 was applied (Bonferroni correction for multiple comparisons).

### Ethics

Ethical approval was obtained from the Hammersmith and Queen Charlotte's & Chelsea Research Ethics Committee (ref 04/Q0406/161). Local Research Governance approval and indemnity, was provided by Imperial College London. All subjects provided written informed consent.

## Results

The clinical details for study participants are given in Table 1.

**Table 1:** Clinical characteristics of PBC patient participants

**Parameter**	Mean (s.d.)
UDCA use	100%
Alkaline phosphatase (ALP)	464 (238.1)
Alanine aminotransferase (ALT)	49.6 (32.1)
Prothrombin time (PT)	12.2 (0.5)
Bilirubin	11.0 (7.9)
PBC‐40 symptoms domain (potential range 7–35)	19.0 (4.3)
PBC‐40 fatigue domain (11–55)	32.4 (11.2)
PBC‐40 cognitive domain (6–30)	15.9 (4.6)
PBC‐40 social & emotional domains (13–65)	34.6 (11.8)
PBC‐30 itch domain (0–15)	5.3 (4.9)

### Magnetisation transfer ratio (MTR)

**Figure 1:**
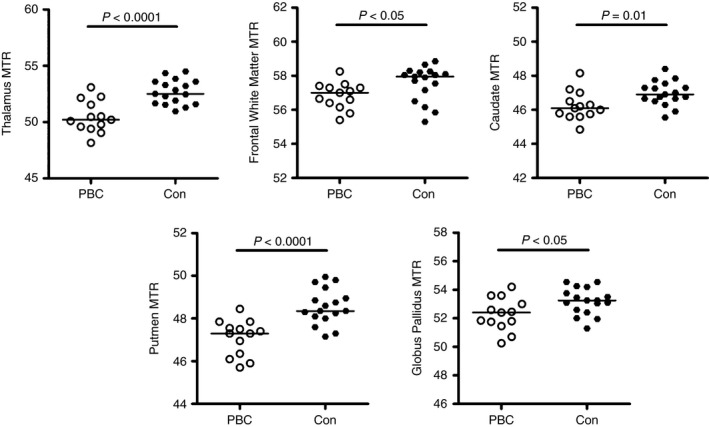
Brain magnetisation transfer ratios (MTR) in PBC patients and normal controls. (a) Thalamus, (b) Frontal White Matter, (c) Caudate, (d) Putamen, (e) Globus Pallidus. All differences were significant at *P* < 0.05 and all remained significant other than globus pallidus following correction for multiple testing.

Magnetisation transfer ratios were significantly decreased in the caudate, putamen and thalamus of precirrhotic PBC patients, compared to the healthy volunteers (Table 2 and Figure 1). The greatest reductions in magnetisation transfer ratios were found in the thalamus (3.9% reduction) and the putamen (3.2% reduction). No statistically significant differences in magnetisation transfer ratios were noted within the group of PBC patients when they were categorised according to self‐reported symptoms on the PBC‐40 assessment tool. There was no correlation between regional brain magnetisation transfer ratios and age in either patients with precirrhotic PBC or healthy controls. There was no significant correlation between the magnetisation transfer ratio data and laboratory biochemical data. In this cohort, there was no association between the pallidal index and manganese levels (*r* = 0.037, *P* = 0.899).

**Table 2:** Regional mean magnetisation transfer ratios (MTR) for precirrhotic PBC patients vs. controls and *P* values of statistical significance using Mann–Whitney test. Regions of the brain showing significant change in PBC patients compared to controls after correction for multiple testing are denoted in bold

Brain region	Mean MTRs (s.d.)		*P* value
	*Control*	*PBC patients*	
Frontal white matter	**57.56 (1.02)**	**56.83 (0.77)**	**0.01**
Caudate	**46.96 (0.72)**	**46.23 (0.84)**	**0.01**
Putamen	**48.56 (0.83)**	**47.09 (0.84)**	**<0.0001**
Globus pallidus	53.19 (0.93)	52.28 (1.15)	<0.05
Thalamus	**52.67 (1.09)**	**50.48 (1.42)**	**<0.0001**

### Diffusion‐weighted imaging (DWI)

**Figure 2:**
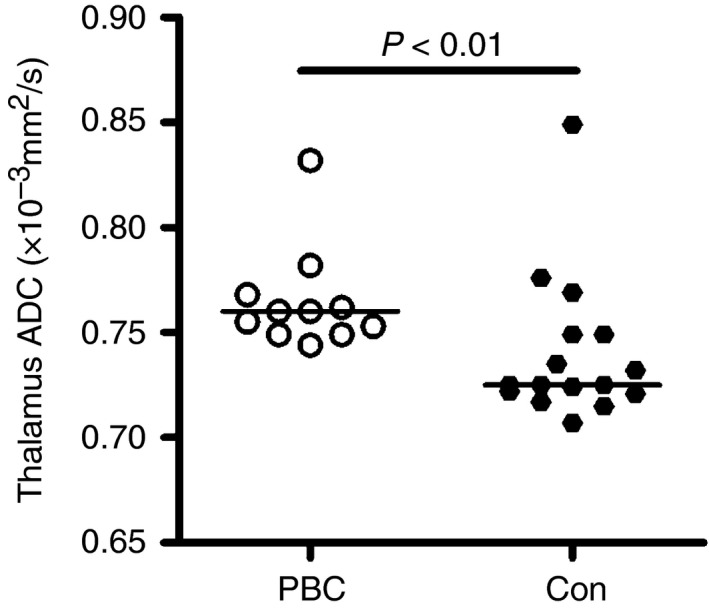
Brain apparent diffusion coefficients (ADC) in PBC patients and normal controls for the thalamus. Difference was significant at *P* < 0.05 and remained significant following correction for multiple testing. No significant differences were seen for other brain areas.

The apparent diffusion coefficient (ADC) was measured in nine brain regions (Table 3 and Figure 2). The apparent diffusion coefficient was significantly increased only in the thalamus of the PBC patients. There were no other brain regions approaching statistical significance, even before correction for multiple comparisons. There was no significant difference in fractional anisotropy (FA), between patients and controls.

**Table 3:** Regional cerebral mean apparent diffusion coefficients (ADC) for precirrhotic PBC patients vs. controls (×10^−3^ mm^2^/s) and *P* values of statistical significance using Mann–Whitney test. Regions of the brain showing significant change in PBC patients compared to controls after correction for multiple testing are denoted in bold

Brain region	Mean ADC ×10^−3^ mm^2^/s (s.d.)		*P* value
	*Control*	*PBC patients*	
Caudate	0.698 (0.03)	0.702 (0.02)	N.S.
Putamen	0.681 (0.02)	0.697 (0.03)	N.S.
Globus pallidus	0.736 (0.07)	0.719 (0.04)	N.S.
Thalamus	**0.740 (0.03)**	**0.765 (0.02)**	**<0.01**

### Proton Magnetic Resonance Spectroscopy (^1^H MRS) & Pallidal Index (PI)

There was no statistically significant difference in the cerebral metabolite ratios in the basal ganglia between precirrhotic PBC patients and healthy controls (Table 4). Furthermore, there was no statistically significant difference in the pallidal index between precirrhotic PBC patients and controls.

**Table 4:** MRS‐measurable metabolite ratios in the basal ganglia in healthy controls and patients with precirrhotic PBC. *P* value denotes level of statistical significance with the Mann–Whitney test

Metabolite ratio	Cerebral metabolite ratios (s.d.)		*P* value
	Controls	PBC	
mI/Cr	0.204 (0.06)	0.315 (0.56)	0.1
Cho/Cr	0.553 (0.13)	0.494 (0.15)	0.3
NAA/Cr	1.64 (0.11)	1.74 (0.29)	0.4

mI, myo‐inositol; Cr, creatine; Cho, choline; NAA, N‐acetyl aspartate.

### Symptom association

**Figure 3:**
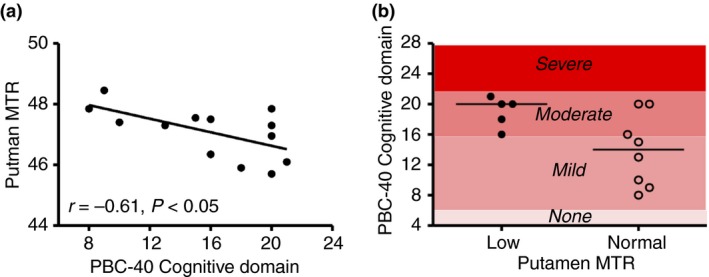
Relationship between putamen magnetisation transfer ratios (MTR) in PBC patients and cognitive symptoms in PBC patients. (a) Correlation between magnetisation transfer ratio level and PBC‐40 Cognitive Domain score. (b) PBC‐40 cognitive domain scores in PBC patients defined as having normal and low Putamen magnetization transfer ratio values (based on a cut‐off of lower level of normal based on data from normal control group. All PBC patients with low putamen magnetisation transfer ratio values had moderate severity cognitive domain scores (the highest severity seen for the cohort of patients enrolled in this study).

Magnetic resonance findings were correlated with the PBC‐40 cognitive and fatigue domains; the domains quantifying CNS‐related symptoms. Of the areas of the brain implicated as abnormal on magnetisation transfer ratios and diffusion‐weighted imaging analysis, association was only seen between cognitive symptom severity and putamen magnetisation transfer ratios (Table 5 and Figure 3). Cognitive symptom impact was relatively low in the study population, compared to the PBC population as a whole (none of the study participants had severe cognitive symptom severity as defined using established cut‐offs).^25^ All the PBC patients with abnormally low putamen magnetisation transfer ratio values (defined using the cut‐off of mean −2s.d. for the normal controls) had moderate cognitive impairment symptoms compared with only 3/8 of the patients with normal putamen magnetisation transfer ratios.

**Table 5:** Associations between fatigue and cognitive symptom severity and degree of abnormality in areas of the brain in PBC showing abnormal magnetisation transfer ratios (MTR) and apparent diffusion coefficients (ADC) values compared to controls. Values in bold denotes *P* < 0.05. (a) magnetisation transfer ratios, (b) apparent diffusion coefficients

Parameter	PBC‐40 cognitive domain score (*r* ^2^)	PBC‐40 fatigue domain score (*r* ^2^)
(a)		
Thalamus	0.08	0.01
Frontal white matter	0.11	0.04
Caudate	0.00	0.03
Putamen	**0.37**	**0.09**
(b)		
Thalamus	0.00	0.03

## Discussion

The findings of this study demonstrate that MR abnormalities are present in the brains of PBC patients from the earliest stages of the disease, within months of disease diagnosis. This finding would support the concept that the disease process in PBC (inflammation, cholestasis or a combination of processes) could cause progressive brain change. The study was not powered to explore the links between brain change and individual symptoms. However, a suggestive association was seen between change in the putamen, an area of the brain playing a key role in learning, and the severity of cognitive symptoms.^27^ The study identifies markers for brain change with the disease, and potentially response markers for therapy aimed at normalising brain function. The findings of this preliminary study need to be replicated in larger cohorts with more detailed information relating to symptom associations. However, they would, if confirmed, provide evidence to support a concept of early aggressive treatment with anticholestatic therapy to reduce the onset of CNS symptoms in this condition.

In the current study, we used multiple, complementary MR imaging modalities (T_1_‐weighted MRI, magnetisation transfer ratios, diffusion‐weighted imaging and ^1^H MRS) in order to explore the full spectrum of potential injury processes in precirrhotic patients. We have previously studied PBC patients with established cirrhosis at 1.5 Tesla (T), finding that magnetisation transfer ratios were significantly reduced,^18^ with increased abnormality levels in more fatigued subjects. The Newcastle group studied 11 patients with PBC with cerebral MRI as part of a study designed to investigate associations between cognitive impairment, autonomic dysfunction and structural brain lesions.^5^ The white matter lesion load correlated with cognitive function, measured by full‐scale Intelligence Quotient (IQ). More recently, Hollingsworth *et al*. studied 30 patients with PBC, measuring magnetisation transfer ratios, T_1_ and T_2_ in the globus pallidus.^20^ They found that magnetisation transfer ratios were negatively correlated with age in early‐stage PBC patients. Forton *et al*. attributed changes in magnetisation transfer ratios to increased manganese deposition.^18^ This may be related to cholestasis that occurs in PBC and thus impaired biliary export of manganese with subsequent sedimentation in areas of high blood flow, such as the basal ganglia. There is biological plausibility to the manganese hypothesis, given established reports of increased manganese deposition in other conditions where T_1_ hyperintensity has been observed, such as welders with occupational manganese exposure^28^ and subjects on long‐term total parenteral nutrition.^29^ Additionally, a strong correlation has been demonstrated between *ante‐mortem* MRI pallidal signal intensity and *post‐mortem* manganese concentrations.^26^ However, reduced magnetisation transfer ratios has also been widely reported in patients with cirrhosis and the etiology suggested to be related to increased brain water content or low‐grade cerebral oedema.^30^ Thus, the etiology of reduced magnetisation transfer ratios and associations with both fatigue and laboratory parameters remains to be confirmed. Neurophysiological approaches such as transcranial magnetic stimulation (TMS) show functional abnormality in regulatory circuits in the CNS.^5^ Animal models of cholestasis, such as the bile duct ligated rodent, show inflammatory change, associated with infiltration of inflammatory cells into the CNS, although, clearly, the potential for cholestasis itself to have neurological effects remains.^31^, ^32^ In the current study, we observed reduced magnetisation transfer ratios in the basal ganglia structures of the thalamus, putamen and head of caudate. The mean apparent diffusion coefficients were only increased in the thalamus. Although serum manganese levels were elevated in the precirrhotic PBC patients, we found no association between the imaging data and blood manganese levels.

MR signal abnormalities in basal ganglia have also been widely reported in patients with any cause of established cirrhosis, most conspicuously on T_1_‐weighted MRI and these were originally thought to be a manifestation of hepatic encephalopathy (HE). Several investigators report correlations between measures of MRI T_1_ hyperintensity in the basal ganglia and blood manganese levels in patients with cirrhosis of any cause, but not always associated with hepatic encephalopathy.^26^, ^33^, ^34^ In the context of established cirrhosis of any cause, reductions in have been attributed to low‐grade cerebral oedema, which is thought to occur in hepatic encephalopathy.^35^ While associations between reduced magnetisation transfer ratios and the Child‐Pugh score have been found by some,^36^ this is not a consistent finding in the literature.^21^, ^37^ Patients with cirrhosis have been shown to have an magnetisation transfer ratio that normalises after liver transplantation.^35^ A further reduction in magnetisation transfer ratio has been induced in patients with cirrhosis by the administration of an amino acid load with a significant change in magnetisation transfer ratio induced in 4 h.^35^ However, in the current study, only patients without cirrhosis, as determined by liver biopsy, were included. Thus, the findings of reduced magnetisation transfer ratios are not a consequence of hepatic encephalopathy, which is supported by the normal ^1^H MRS for the cohort.

In diffusion‐weighted imaging, the mean apparent diffusion coefficients in the thalamus was significantly increased, but in the other measured brain regions did not approach statistical significance, even before correcting for multiple comparisons. While one might intuitively expect diffusion‐weighted imaging measures to be abnormal in the same regions as MT, as both are affected by brain water content, there is evidence in the multiple sclerosis literature that there is often no correlation between magnetisation transfer ratios and diffusion‐weighted imaging measures.^38^, ^39^ This reinforces that these two MR modalities are independent of each other, quantifying different effects within a region of interest. The classical biological interpretation of the increased apparent diffusion coefficients in the thalamus would be the presence of ‘vasogenic’ or extracellular oedema.^39^, ^40^, ^41^, ^42^ However, it must be appreciated that effects from proteins, phospholipids and extracellular matrix may affect the diffusion of water molecules,^43^ rather than just an increase in the amount of extracellular water. Indeed, manganese deposition within the thalamus could result in alteration of the cell membrane permeability, thereby affecting the water diffusivity and that accumulation of manganese within the extracellular matrix, or intracellularly, may affect the apparent diffusion coefficients. Due to the fact that the increased apparent diffusion coefficients were only found in one brain region, it is possible that other areas of the brain may have yielded significant results. However, given that this was a pilot study, insufficient numbers of subjects may have been contributory.

The finding of normal ^1^H MRS in this cohort of PBC patients, of whom more than 50% reported symptoms of moderate or severe fatigue, is interesting. ^1^H MRS has been found to be abnormal in patients with impaired quality of life attributed to liver disease. In patients with cirrhosis and hepatic encephalopathy, reduced basal ganglia choline/creatine (Cho/Cr) and myo‐inositol/creatine (mI/Cr) ratios have been widely reported.^44^, ^45^, ^46^ Elevated Cho/Cr ratios in the basal ganglia have been reported in patients with mild hepatitis C without cirrhosis^47^ and were associated with impaired psychometric performance. Thus, the absence of neurospectroscopic abnormalities may suggest an alternative mechanism to that which affects the quality of life of patients with cirrhosis or mild hepatitis C.

This is the first neuroimaging MR study specifically to look at precirrhotic PBC. We sought to identify whether there may be CNS change early in the disease and our findings would confirm that there is. Larger scale, and in particular linear studies, will be needed to explore the relationship of this change to symptoms and its response to therapies such as UDCA and OCA. The presence of brain change so early in the disease process would, however, suggest that the current step‐up approach to therapy in which treatment change follows failure of a therapy type may allow the progressive accumulation of brain injury whilst waiting for adequate therapeutic response.

## Authorship

*Guarantor of the article*: Professor David EJ Jones.

*Author contributions*: The study was conceived by RWC, DEJJ and SDTR and conducted by VPBG, MMEC, MWA, NP and JAF. Data analysis was performed by VPBG, LS and ABB with interpretation by JKD, JUK, ADW, GJA, DEJJ and SDTR. The manuscript was written by VPBG, JKD, JUK, MMEC, GFM, DEJJ and SDTR.

All authors contributed to and approved the final manuscript.
